# The CnuK9E H-NS Complex Antagonizes DNA Binding of DicA and Leads to Temperature-Dependent Filamentous Growth in *E. coli*

**DOI:** 10.1371/journal.pone.0045236

**Published:** 2012-09-13

**Authors:** Sang Hoon Yun, Sang Chun Ji, Heung Jin Jeon, Xun Wang, Si Wouk Kim, Geunu Bak, Younghoon Lee, Heon M. Lim

**Affiliations:** 1 Department of Biology, College of Biological Sciences and Biotechnology, Chungnam National University, Taejon, Republic of Korea; 2 Department of Environmental Engineering, Pioneer Research Center for Controlling of Harmful Algal Blooming, Chosun University, Gwangju, Republic of Korea; 3 Department of Chemistry, KAIST, Daejeon, Republic of Korea; University of Massachusetts Medical School, United States of America

## Abstract

Cnu (an OriC-binding nucleoid protein) associates with H-NS. A variant of Cnu was identified as a key factor for filamentous growth of a wild-type *Escherichia coli* strain at 37°C. This variant (CnuK9E) bears a substitution of a lysine to glutamic acid, causing a charge reversal in the first helix. The temperature-dependent filamentous growth of *E. coli* bearing CnuK9E could be reversed by either lowering the temperature to 25°C or lowering the CnuK9E concentration in the cell. Gene expression analysis suggested that downregulation of *dicA* by CnuK9E causes a burst of *dicB* transcription, which, in turn, elicits filamentous growth. *In vivo* assays indicated that DicA transcriptionally activates its own gene, by binding to its operator in a temperature-dependent manner. The antagonizing effect of CnuK9E with H-NS on DNA-binding activity of DicA was stronger at 37°C, presumably due to the lower operator binding of DicA at 37°C. These data suggest that the temperature-dependent negative effect of CnuK9E on DicA binding plays a major role in filamentous growth. The C-terminus of DicA shows significant amino acid sequence similarity to the DNA-binding domains of RovA and SlyA, regulators of pathogenic genes in *Yersinia* and *Salmonella*, respectively, which also show better DNA-binding activity at 25°C.

## Introduction

Cnu (an OriC-binding nucleoid protein) associates with H-NS (a histone-like nucleoid structuring protein).The Cnu-H-NS protein complex binds to a specific sequence at the origin of chromosomal DNA replication in *Escherichia coli* [1]. Cnu has 71 amino acids and 39% amino acid identity with the Hha protein, also known to complex with H-NS to downregulate the hemolysin gene of *E. coli* [2]. The Cnu protein, formerly identified as YdgT, was first described as a homolog of Hha and was reported to complex with the StpA protein, a homolog of H-NS, in *E. coli* [3]. These small H-NS binding proteins are found in enterobacteria. In Yersinia, YmoA [4] and a list of other proteins all appear to share the ability to bind H-NS and regulate gene expression at the transcriptional level [5].

H-NS is a non-specific DNA binding protein. However, many reports [6]–[8] have raised the possibility that H-NS binds to DNA in a sequence-specific manner by binding small accessory proteins, resulting in protein complexes involved in gene regulation. Much remains to be discovered about the physiological significance of the H-NS complexes; NMR structures of the Cnu and Hha proteins are available [9], [10] and the structures of the N-terminal (dimerization) and C-terminal (DNA binding) domains of H-NS are known [11]–[13]. A recent crystallographic study showed the structural basis for H-NS oligomerization [14], and the oligomerization of H-NS on DNA leads the formation of the two specific clusters of H-NS on the chromosomal DNA of *E. coli* [15]. However, the structure of the Cnu-H-NS complex and the DNA binding mode of the complex are not known. During the course of experiments on the interaction between Cnu and H-NS, we obtained a Cnu mutant, CnuK9E, which elicits temperature-dependent filamentous growth in *E. coli*.

**Figure 1:**
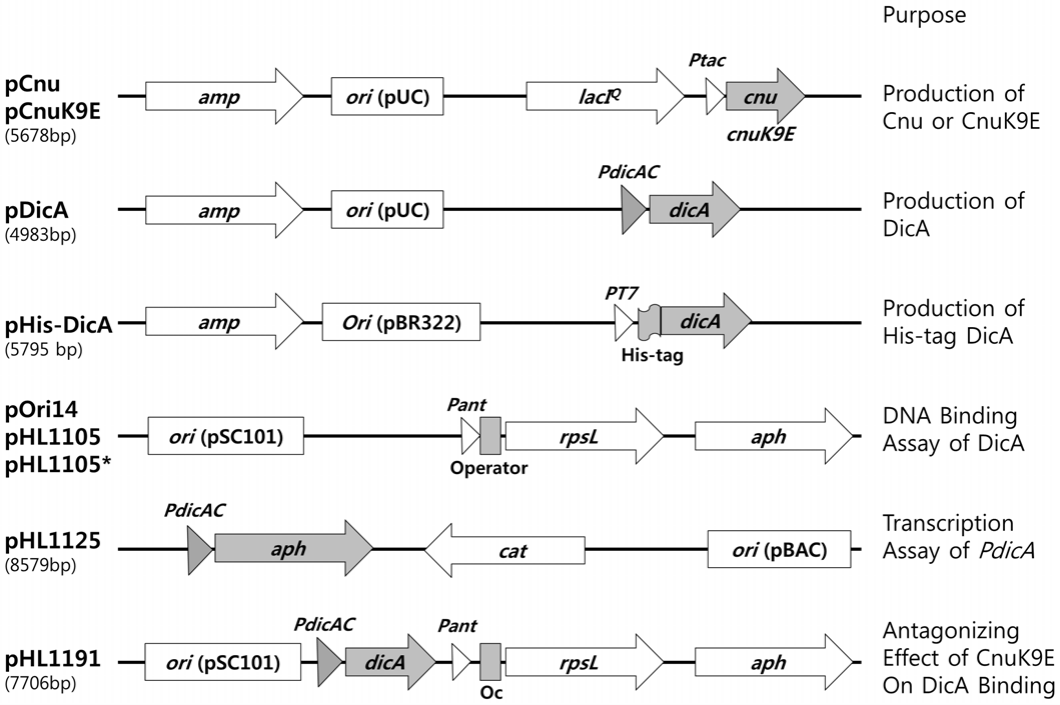
Plasmids used in this study. Plasmids are schematically presented along with their names, size in base pairs (bp), and purpose. Genes are depicted as arrows pointing in the direction of transcription. The origin of DNA replication (*ori*) is presented in a box with the conventional name of the plasmid. pOri14, pHL1105, and pHL1105* have the same sequence except the operator. The following sequences are present at the operator; 5′ATGATCGGTGATCCTG for pOri14, 5′TTGTTAGTCATAACTAACAA for pHL1105, and 5′TTGTTAGTCATAACTCACAA for pHL1105*. pOri14 has 7219 bp, and both pHL1105 and pHL1105* have 7216 bp each.

Here, we describe the molecular basis of the temperature-dependent filamentous growth caused by CnuK9E. Our data indicate that when CnuK9E is expressed, DicA production is reduced to one-tenth the normal level, and expression of DicB, a cell division inhibitor [16], increased 3,700 fold, uniquely at 37°C. *In vivo* assays suggested that CnuK9E in complex with H-NS antagonizes DicA binding to its own gene promoter, resulting in a 90% reduction of DicA production at 37°C.

**Figure 2:**
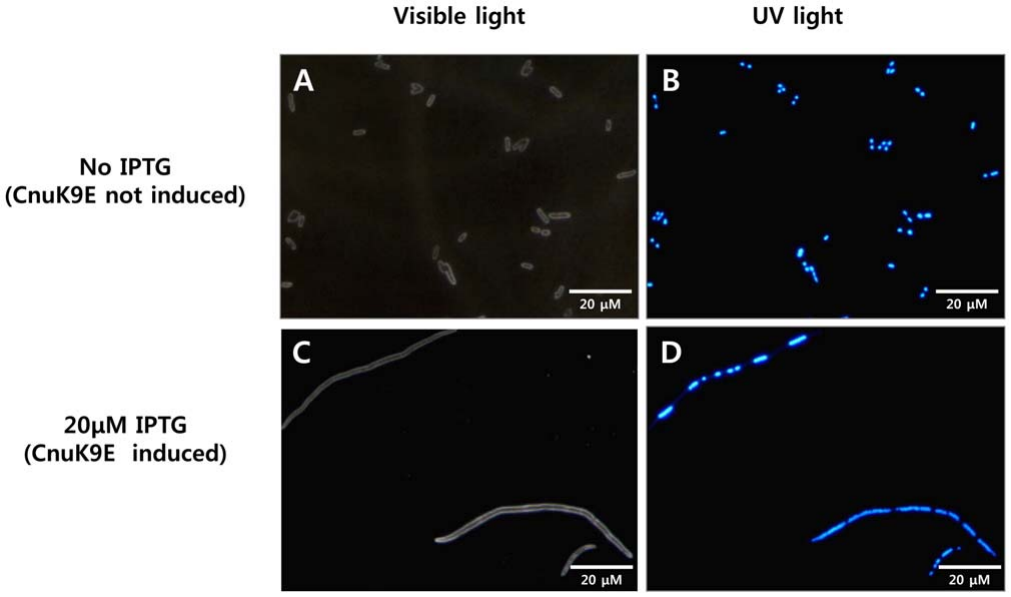
Filamentous growth of MG1655/pCnuK9E. MG1655 harboring pCnuK9E was grown in LB liquid medium at 37°C. Cells were stained with Hoechst 33342 dye. Cells grew normally when CnuK9E was not induced (A). When the same microscopic field was observed under UV light, each cell had one or two discrete nuclei (B). When CnuK9E was induced, however, the cells adopted a filamentous form (C). MG1655 cells harboring pCnu grew normally when Cnu was overexpressed to the same level of CnuK9E (data not shown). The same cells in C had several discrete nuclear regions when observed under UV light (D).

## Materials and Methods

### Bacterial strains and media

*E. coli* strains MG1655 and HB101 (F- *mcrB mrr hsdS20 recA13 leuB6 ara-14 proA2 lacY1 galK2 xyl-5 mtl-1 rpsL20(SmR) glnV44 λ-*) and their derivatives were used throughout this study. A derivative of BL21(DE3)/pLysS, BL21(DE3)*hns*/pLysS was used for the preparation of proteins. Deletion of specific genes from these strains was performed by precisely removing the target gene following the procedure of Yu *et* *al.,* [17]. LB (tryptone 10 g, yeast extract 5 g, NaCl 10 g per liter water) was used for liquid culture and LB agar plates were used for colony growth on solid medium [18].

**Figure 3:**
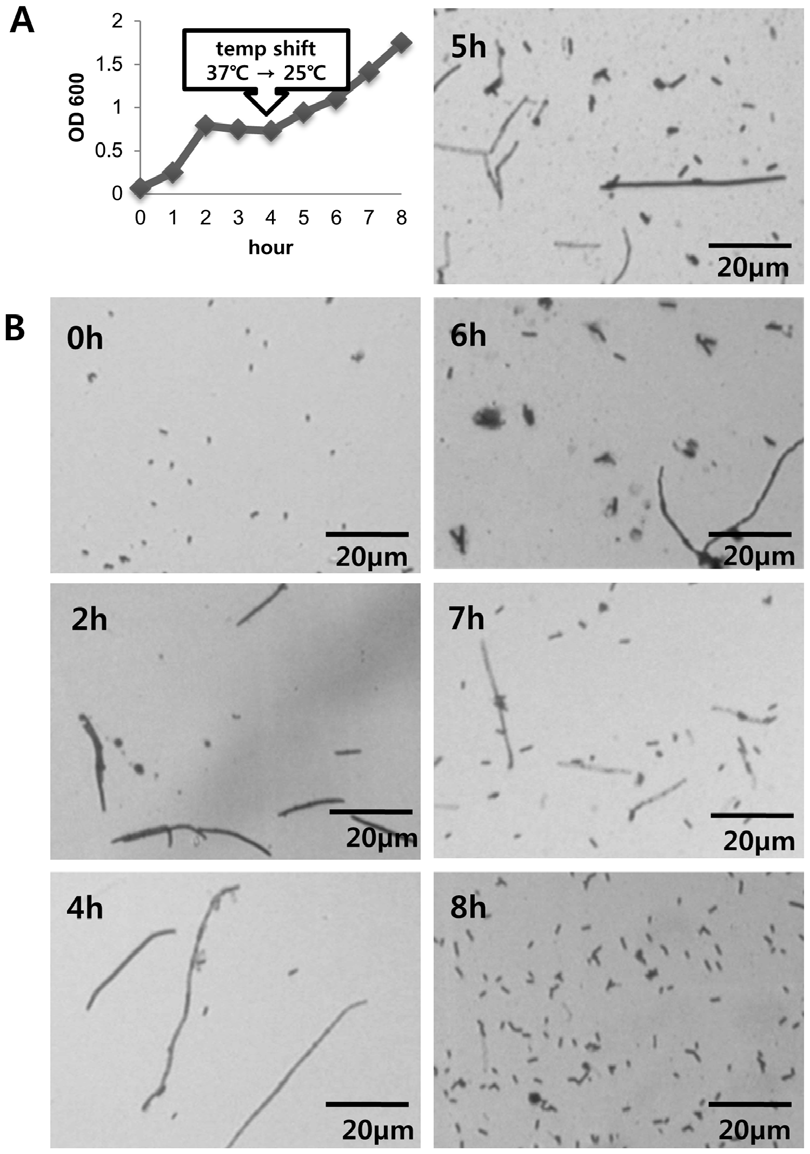
The filamentous growth of MG1655/pCnuK9E can be reversed to normal growth. MG1655/pCnuK9E cells grown in LB liquid medium at 37°C became filamentous 4 h after induction of CnuK9E (B, 4 h). When the culture was transferred to 25°C and continued, cells resumed normal growth 4 h after the temperature shift (B, 8 h). The cell growth of this experiment is presented as OD_600_ vs. growth time (A).

### Plasmid construction

The *cnu* and *dicA* genes were PCR-amplified with primer pairs having specific restriction sites of at their ends, and cloned into the corresponding restriction sites of pHL355 [1] to make pCnu or pDicA. This method of cloning has been used throughout this study. Primers used in this study are listed in the Table S1. Synthetic DNA fragments of Ori14-up, Oc-up, Oc*-up, Ori14-down, Oc-down, and Oc*-down were hybridized to make double stranded DNA of Ori14, Oc, and Oc* sequences, and cloned into the SmaI site of pHL343 [1], to create pHL562, pHL1104, and pHL1104*, respectively(*:single base change). The EcoRI-EcoRV restriction fragment of pHL204 [19] was replaced with the EcoRI-EcoRV restriction fragment of pHL562, pHL1104, or pHL1104* completing the assembly of the substrate plasmids, pOri14, pHL1105, and pHL1105*. To make pHL1124, a PCR-amplified kanamycin resistance gene was inserted into the BamHI-ScaI site of pCC1BAC(Epicentre). To make pHL1125, PCR-amplified *PdicAC* (103 bp) was inserted into the EcoRI-BamHI site of pHL1124. The *dicA* gene, including the promoter (*PdicAC*), was PCR-amplified and cloned into the EcoRV site of pHL1105, making pHL1191. Cloned DNA sequences were confirmed by DNA sequencing.

**Figure 4:**
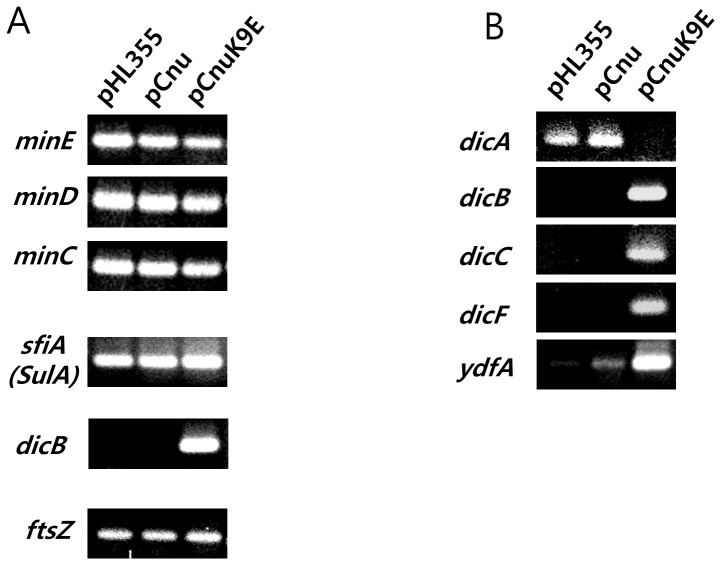
RT-PCR analysis of the expression of genes involved in cell division. Expression levels of genes involved in septal ring formation (A) and known to be repressed by *dicA* (B) were analyzed in MG1655 cells harboring pHL355 (vector control), pCnu (expressing WT Cnu), or pCnuK9E (expressing CnuK9E) at 37°C.

### Cnu random mutagenesis and screening for streptomycin (Sm)-sensitive colonies

A random mutagenesis of *cnu* was performed using error-prone PCR. The *cnu* gene was amplified in a PCR reaction containing 10 ng of pCnu, 0.5 µM of each primer (pHL355-RM-F, pHL355-RM-B), 5 U Taq DNA polymerase (Bioneer, Daejeon, Korea), dNTP mix (1 mM dCTP, 1 mM dTTP, 0.2 mM dATP, 0.2 mM dGTP, final concentration), and 1× error-prone PCR buffer [10 mM Tris-HCl (pH8.0), 50 mM KCl, 7 mM MgCl_2_, 0.01% gelatin (w/v)] under the following conditions: an initial denaturation step at 95°C for 3 min, followed by 40 cycles of 30 s of denaturation at 94°C, 30 s of hybridization at 50°C, and 30 s of elongation at 72°C. The PCR product was purified using the Qiaquick PCR purification kit (Qiagen, Valencia, CA) and ligated into EcoRI/BamH1-digested pHL355. The ligation was performed overnight at 25°C and introduced into HB101△*cnu* △*hha*/pOri14 by electroporation. The transformed cells were plated on LB agar plates containing ampicillin (Amp;100 μg ml^−1^) and kanamycin (Kan; 50 μg ml^−1^), and incubated at 37°C overnight. The next morning, transformants were stabbed simultaneously onto LB agar plates containing Amp, Kan, Str (100 μg ml^−1^ ), and 20 µM IPTG (isopropyl-β-D-thiogalactopyranoside), and on the same LB agar plates lacking Sm, and incubated at 37°C. We screened over 2000 colonies and found 89 Sm-sensitive colonies. CnuK9E was identified independently three times. The pCnu plasmid was isolated from the Sm-sensitive colonies, and mutations were identified by DNA sequencing of the *cnu* gene (Solgent, Taejon, Korea).

**Figure 5:**
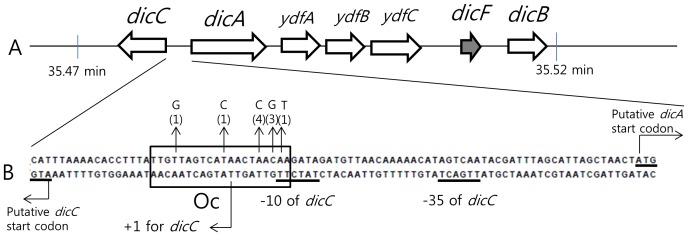
Relative location of *dic* genes on the *E. coli* chromosome and DNA sequence of the promoter region of the *dicA* and *dicC* genes (*PdicAC*). (A) A portion of the Qin prophage in the *E. coli* genome between 35.47 and 35. 52 min is shown with genes involved in cell division inhibition [27]. Arrows indicate the direction of transcription and relative size of the genes. The *dicF* gene produces RNA only [30]. (B) DNA sequence of the promoter region of the *dicA* and *dicC* genes is shown. The putative DicA-binding site (Oc) is boxed. The nucleotide changes detected in a few cloned *PdicAC* DNA fragment are shown above the arrows with numbers in parentheses that indicate the number of incidents. A putative −10 and −35 promoter sequence of the *dicC* gene is underlined. The transcription initiation site of the *dicC* gene is indicated as +1 for *dicC* (Fig. S3). The promoter sequence for the *dicA* gene is not obvious with DNA sequence information only.

**Table 1:** Relative concentration of *dic* transcripts*.

Host strain	MG1655				MG1655*hns*			
Plasmid	pHL355		pCnuK9E		pHL355		pCnuK9E	
Temp	25°C	37°C	25°C	37°C	25°C	37°C	25°C	37°C
*dicA*	1	1.7	0.7	0.1	1	0.8	0.61	0.55
*dicC*	1	1.5	48.2	704	1	0.59	44.8	24.5
*dicB*	1	2.2	49.2	3743	1	1.28	44.2	78.7

* These values were measured by three independent experiments. Average values are presented.

### Visualization of DNA in MG1655/pCnuK9E cells

MG1655/pCnuK9E cells were grown in 3 ml LB/Amp overnight without IPTG to keep *cnuK9E* expression repressed at 37°C. The next morning, a 1∶100 dilution of the overnight culture was made in 50 ml LB/Amp (for normal growth) and in LB/Amp/20 µM IPTG (for filamentous growth). The cultures were incubated at 37°C with shaking at 250 rpm. A 1-ml aliquot of each culture was collected. Cells were pelleted and washed twice with 1 ml phosphate-buffered saline (PBS) and resuspended in 500 μl PBS. Hoechst33342 (0.5 μl, 1 mg ml^−1^, Sigma-Aldrich, St. Louis, MO) was added to stain the nucleic acid. The sample was incubated for 10 min at 25°C and then washed four times with 500 μl PBS. Cells were visualized and photographed using a Leica DM5000 B microscope (Wetzlar, Germany).

**Figure 6:**
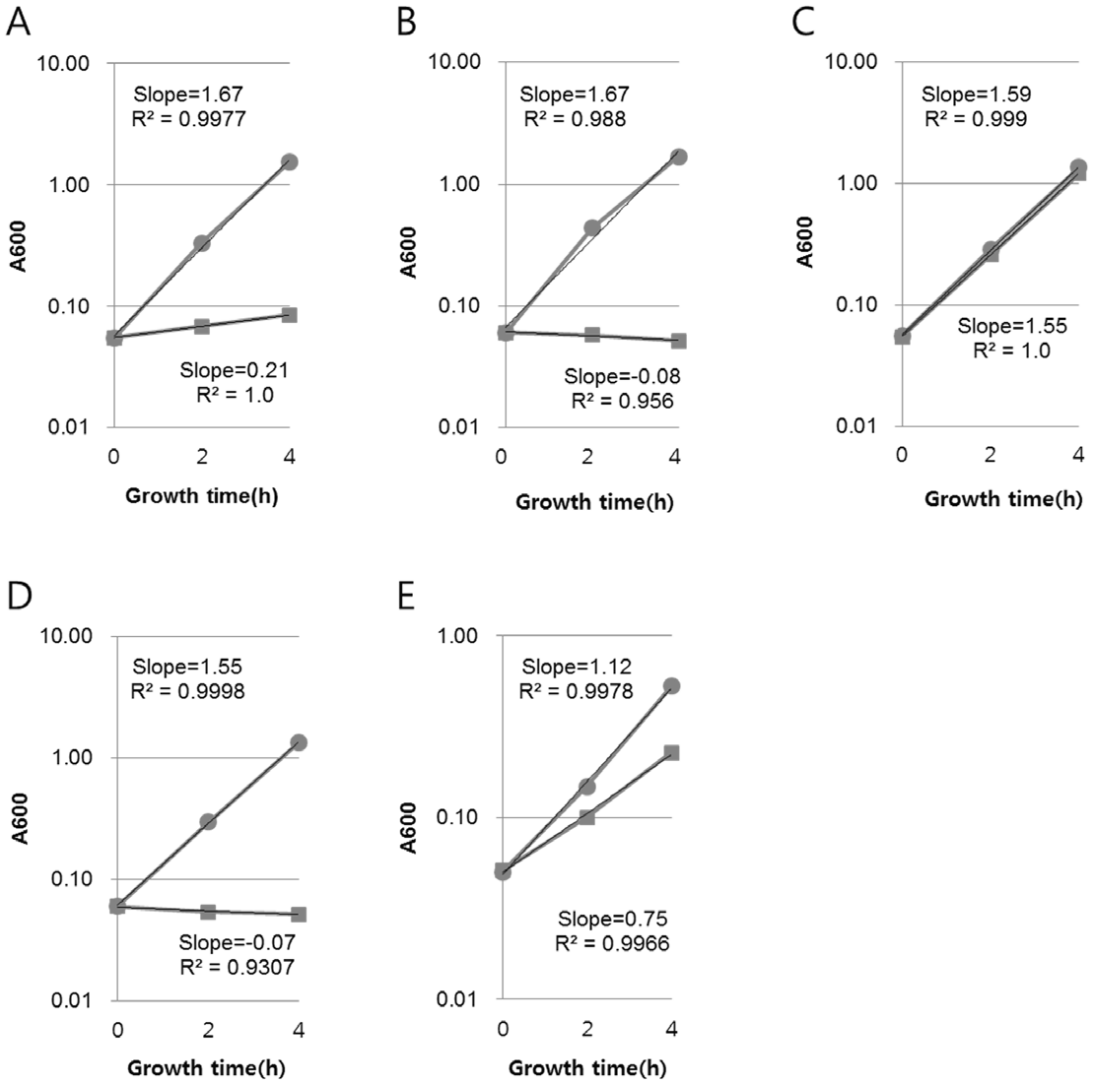
*In vivo* measurements of DicA binding to Oc. *In vivo* DNA-binding activity of DicA to Oc was measured by the growth rate of the host cell. The growth rate at 37°C of the strains HL100/pHL1105 (A), HL100*△dicA*/pHL1105 (B), HL100*△dicA*/pHL1105/pDicA (C), and HL100/pHL1105* (D) was measured in LB medium (circle) and in LB containing streptomycin (rectangle). Growth rate was determined from the slope of the fitted curve of the bacterial growth measured by OD_600_. The regression coefficient for the fittingindicated that all the fittings are statistically significant. The growth rate of HL100/pHL1105 was also measured at 25°C (E).

### RNA preparation and mRNA quantification

Cells were grown in 50 ml LB broth to an OD_600_ of 1.0 as described above. Total RNA from the culture was prepared, as described in [20]. Total RNA (2 μg) was reverse transcribed in a 20-μl reaction containing 4 U Omniscript Reverse Transcriptase (QIAGEN), 0.5 mM each dNTP, 10 µM random nonamer (Takara Bio, Shiga, Japan), and 10 U RNasin. PCR amplification of a specific cDNA was performed in a 20-μl reaction using 1 μl cDNA [20]. Real-time qPCR was performed in a 10-μl reaction containing 5 μl iQTM SYBR Green Supermix (Bio-Rad, Hercules, CA), 3.6 μl nuclease-free water, 0.2 μl each primer (10 µM), and 1 μl cDNA template under the following conditions: an initial denaturation step at 95°C for 3 min; 40 cycles of 10 s of denaturation at 94°C, 20 s of hybridization at 56°C, and 15 s of elongation at 72°C (Bio-Rad). Primers for real-time qPCR are listed in Table S1.

**Table 2:** Growth Ratio.

Host strain	Substrate Plasmid	Temp	Growth rate in LB	Growth rate in LB/Str	Growth Ratio a(GR)
HL100	pHL1105	37°C	1.67	0.21	0.13
HL100*dicA*	pHL1105	37°C	1.67	–0.08	–0.05
HL100*dicA*/pDicA	pHL1105	37°C	1.59	1.55	0.97
HL100	pHL1105*	37°C	1.55	–0.07	–0.05
HL100	pHL1105	25°C	1.12	0.75	0.67

a The ratio of the growth rates between the two media (LB-Str/LB) measures the degree of DicA binding to Oc, higher the better.

### Measurement of the Growth Ratio (GR)

DicA binding activity was measured in vivo using the ratio of growth rates of cells harboring pHL1105 without/with Sm (Growth Ratio, GR). The overnight culture of each strain harboring pHL1105 grown at 25°C or 37°C in LB with proper antibiotics was diluted to an OD_600_ of 0.05 in LB with proper antibiotics and IPTG, if necessary. The culture was incubated at the desired temperature with continuous shaking (200 rpm). For the GR measurement, the OD_600_ was measured every 2 h.

**Figure 7:**
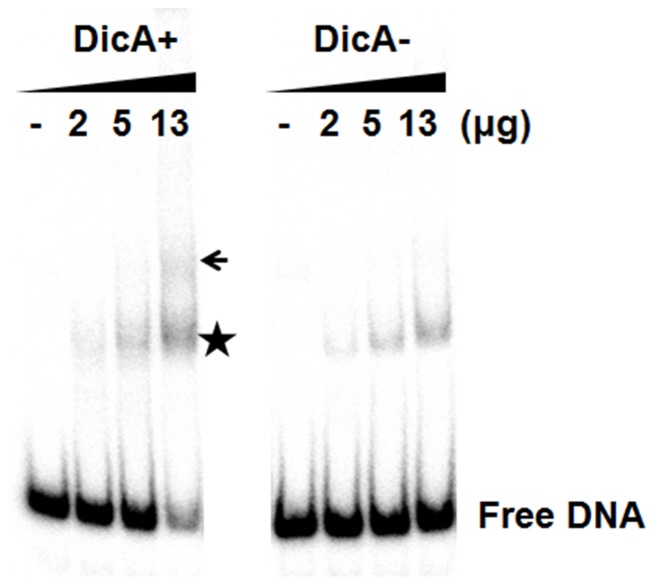
*In vitro* measurements of DicA binding to Oc. Electrophoretic mobility shift assay was performed with radio-labeled 90-bp DNA containing Oc. Soluble protein extracts from BL21 (DE3) cells harboring pHis-DicA (Fig. 1) of 0, 2, 5, or 13 μg were used in the binding assay (Fig. S2). DicA + indicates DicA induced with IPTG. DicA− indicates no DicA induction. The binding reaction was incubated for 20 min at 25°C, and subjected to electrophoresis on a 5% polyacrylamide gel.

**Table 3:** IC_50_ kan at 25°C and 37°C.

		IC_50_ kan^a^ (µg ml^−1^)	
Host cell	Substrate plasmid	25°C	37°C
HL100	pHL1125	94±8.1	73±3.1
HL100	bpHL1124	3.8±0.3	4.3±0.2
HL100*dicA*	pHL1125	3.5±0.5	4.4±0.6
HL100*dicA*/pDicA	pHL1125	40±0.6	36±1.0

a Half maximal inhibitory concentration for kanamycin. Higher value means More *dicA* transcription.

b pHL1124 has no *PdicAC.*

### IC50 measurement

To determine the concentration of the antibiotic kanamycin (Kan) required for 50% inhibition of bacterial growth (IC50) [21], the overnight culture of each strain harboring pHL1125 was diluted to an OD_600_ of 0.05 in 5 ml LB containing chloramphenicol and different concentrations of Kan. After a 3 h shaking incubation (200 rpm) at 25°C or 37°C, the OD_600_ was measured. The Kan concentration that gave rise to half the OD_600_ of cultures of the Kan-minus control was taken as IC50 [21].

**Figure 8:**
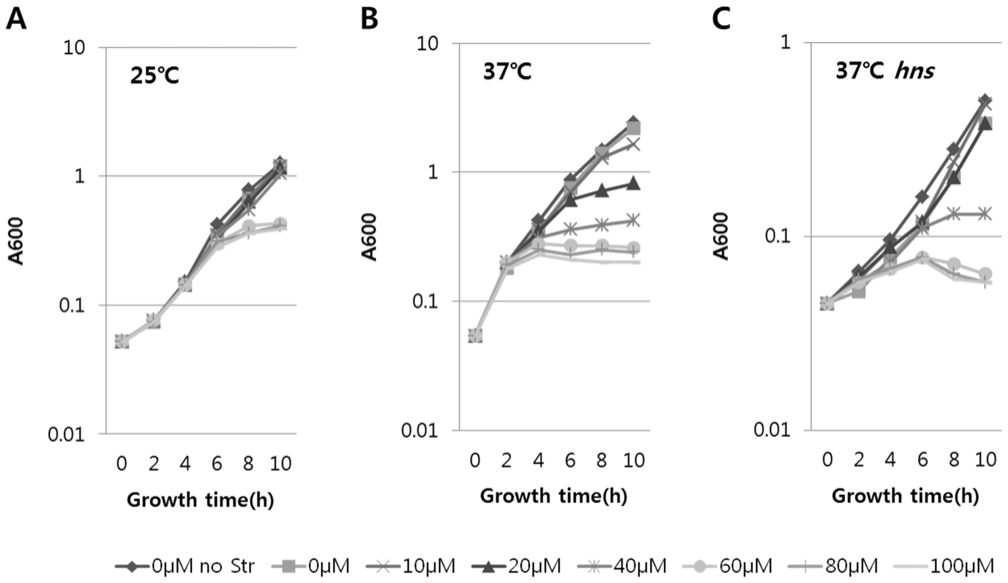
*In vivo* measurement of the antagonistic effect of CnuK9E on DicA binding to Oc. The DNA binding activity of DicA to Oc was measured with different concentrations of IPTG, resulting in different concentrations of CnuK9E. Growth of HL100/pHL1191/pCnuK9E was measured at 25°C (A) and 37°C (B). The antagonizing effect of CnuK9E was also measured in HL100*△hns*/pHL1191/pCnuK9E (C) where H-NS is absent. The symbol for each IPTG concentration is presented below the graphs.

## Results and Discussion

### Selection of a cnuK9E mutant strain

The initial purpose of these experiments was to understand the mechanism of interaction between the Cnu and H-NS proteins by isolating Cnu variants unable to interact with H-NS [22]. The selection criteria for the Cnu variants were based on our previous *in vivo* results, and were performed at 37°C. H-NS, a non-specific DNA binding protein, binds to a specific sequence of *oriC* once complexed with Cnu [1]. The principle of the in vivo DNA binding assay performed in this study is the following; An *E. coli* strain, HB101 harboring the *rpsL20* allele in the chromosome is resistant to streptomycin (Sm). Once HB101 harbors the wild-type *rpsL* gene in a plasmid such as pOri14 (Fig. 1), the host cell becomes sensitive to Sm. DNA binding of a protein or protein complexes to a specific sequence of *oriC* (Ori14, Fig. 1), which resides at an operator in the artificial *rpsL* operon of the plasmid pOri-14, causes host HB101cells to regain streptomycin-resistanance [1], [19].

**Figure 9:**
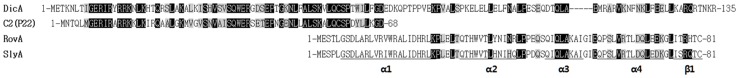
Amino acid alignments of DicA with functionally and structurally homologous proteins. The N-terminal part of DicA lines up well with the N-terminus of the C2 (GenBank: NP_059606.1) protein from the *Salmonella* phage P22. The C-terminal part of DicA shares significant amino acid sequence homology with the N-termini of RovA (GenBank: AAK01704.1) of *Yersinia* and SlyA (GenBank: AAL55673.1) of *Salmonella*. Identical amino acids with DicA are marked in black. The winged helix DNA-binding domain is underlined with the secondary structure labeled. The amino acid sequences are taken from the GenBank database, and the sequence comparison algorithm BLAST [33] was used.

We used two plasmids for the screen, a high copy-number plasmid from which the Cnu protein could be induced and over-expressed (pCnu, Fig. 1), and a low copy-number plasmid encoding the *rpsL* operon (pOri14, Fig. 1). Cells in which both *cnu* and *hha* genes are deleted (HB101*△cnu△hha*), and that are co-transfected with these two plasmids, produce Sm-resistant colonies only when the Cnu protein is induced. In a control experiment with the strain HB101*△cnu△hha△hns*, in which all *cnu, hha,* and *hns* genes are deleted, cells were Sm-sensitive even when the Cnu protein was over-expressed. These results demonstrate that both Cnu and H-NS are required to make the host cells resistant to Sm, presumably because the Cnu-H-NS complex binds to the operator DNA (Ori14), which, in turn, represses transcription of the *rpsL* gene. We randomly mutagenized the *cnu* gene in pCnu and screened for Sm-sensitive colonies (see Materials and Methods).

Sm-sensitive colonies were screened at 37°C and DNA sequencing of the *cnu* genes from the pCnu plasmids revealed a Cnu variant, CnuK9E (lysine to glutamic acid substitution at residue 9), in which expression of CnuK9E caused filamentous growth of the host cells only at 37°C.

### CnuK9E causes temperature-sensitive filamentous growth of *E. coli*

The induction of CnuK9E expression in HB101*△cnu△hha* cells harboring pCnuK9E and pOri14 caused the cells to grow in a filamentous form. Surprisingly, the induction of CnuK9E in HB101*△cnu△hha* cells harboring only pCnuK9E (without pOri14) also resulted in filamentous growth. Therefore, we wondered how could we have selectedHB101*△cnu△hha* cells harboring pCnuK9E and pOri14 were selected in the screen for Sm-sensitivity initially. Growth experiments with different combinations of *E. coli* strains and the two plasmids showed that any *E. coli* strain harboring the pCnuK9E plasmid exhibited filamentous growth in liquid culture and failed to form colonies on agar plates only when the CnuK9E protein was over-expressed from the plasmid. These results suggest that it is the CnuK9E protein that makes *E. coli* cells adopt the filamentous morphology. CnuK9E was initially selected because the cells could not form colonies on solid medium at 37°C, not because the host cells became Sm-sensitive. A wild-type *E. coli* strain, MG1655, harboring pCnuK9E (MG1655/pCnuK9E) grew normally if CnuK9E was not induced, but exhibited filamentous growth when CnuK9E was induced in liquid medium (Fig. 2). The average length of a single filamentous cell was 30 to 120 µm. Staining of the nuclear region revealed that each filamentous cell had dispersed but discrete nuclei (Fig. 2), suggesting that cytokinesis did not occur. The more dispersed appearance of nuclei in CnuK9E expressed cells than normal cells, perhaps indicates that overexpression of CnuK9E disorganize the chromosome compacted by H-NS [15]. Nonetheless, at 25°C *E. coli* cells harboring pCnuK9E grow normally, suggesting that the filamentous growth phenotype caused by CnuK9E was temperature sensitive (data not shown).

### The switch to filamentous growth is reversible

We then asked whether the filamentous growth observed at 37°C could be restored to normal if the temperature was lowered to 25°C. MG1655 cells harboring pCnuK9E were grown overnight in lysogeny broth (LB, [18] containing ampicillin at 37°C, and then a 1∶100 dilution was made with fresh LB containing ampicillin and IPTG to induce CnuK9E expression. The fresh culture was grown at 37°C. Cells grew to an optical density at 600 nm (OD_600_) of 0.8 in the first 2 h, but the OD_600_ did not change over the next 2 h (Fig. 3A). Microscopic observation of the cells during the first 4 h of growth showed that cells started to become filamentous at 2 h, and at 4 h cells were fully filamentous (Fig. 3B). At 4 h, we shifted the culture to 25°C and the OD_600_ of the culture started to increase immediately. Microscopic observation indicated that the cells started to divide normally 1 h after and regained the normal *E. coli* cell shape 4 h after the temperature shift down (Fig. 3B).

We also attempted to reverse the filamentous growth by removing IPTG from the liquid medium, thereby depleting CnuK9E from the cells. MG1655/pCnuK9E cells were induced to the filamentous form, as described above. At 4 h, the cells were pelleted by centrifugation, washed twice with fresh LB, and resuspended in fresh LB/Amp (without IPTG). Growth was allowed to continue at 37°C. Microscopic observation indicated that cells started to regain a normal shape and growth 3 h after removal of IPTG (data not shown). Taken together, these results demonstrated that CnuK9E is the key factor for the filamentous growth, and that the filamentous growth occurs at 37'C but not at 25'C.

### H-NS is required for CnuK9E to induce filamentous growth

Because Cnu forms a protein complex with H-NS [1], [3], we next asked whether CnuK9E required H-NS to elicit filamentous growth. We deleted the *hns* gene from MG1655 (MG1655*△hns*) and determined whether pCnuK9E/MG1655*△hns* exhibited filamentous growth when CnuK9E was induced at 37°C. MG1655*hns* was shown to grow normally in liquid culture and could form single colonies on agar plates even in the presence of CnuK9E, suggesting that CnuK9E requires H-NS to induce filamentous growth at 37°C. Then, we asked whether CnuK9E could form a complex with H-NS. We noticed during the His-tag based purification of Cnu that H-NS protein eluted with His-tagged Cnu from the nickel-affinity column [1]. We showed that H-NS protein also co-eluted with CnuK9E (Fig. S1), suggesting that CnuK9E forms a complex with H-NS, as does Cnu.

We also analyzed the growth of pCnuK9E/MG1655*△stpA*, an *E. coli* strain with a deletion in the StpA gene that encodes an H-NS homolog [23]. The results show that MG1655*△stpA* exhibited filamentous growth at 37°C when CnuK9E was expressed (data not shown), suggesting that StpA is not required for the Cnu-K9E induced filamentous growth. Taken together, these data suggest that CnuK9E could form a protein complex with H-NS and that both proteins are required to elicit filamentous growth in *E. coli* at 37°C.

### Transcriptional downregulation of *dicA* is likely the cause of the filamentous growth

We next examined the molecular mechanism of filamentous growth induced by CnuK9E. We rationalized that CnuK9E gained a new function in gene regulation during cytokinesis and a literature search of cytokinesis in *E. coli* suggested several possibilities. During cytokinesis, a series of proteins must be expressed to form the septal ring at the right time and place (reviewed in [24]. We investigated the expression of all known genes involved in FtsZ protein-mediated septal ring formation at the non-permissive temperature 37°C, from *ftsZ* to *amiC,* as described previously [24]. RT-PCR analysis was performed to measure the expression of these genes in a wild-type strain (MG1655) harboring the plasmids pHL355, identical to pCnu but lacking the *cnu* gene, pCnu, or pCnuK9E. The expression levels of all genes tested were identical in each of the three genetic backgrounds (data not shown), suggesting that CnuK9E is not likely to be involved in regulating the expression of genes necessary for the FtsZ-mediated septal ring formation. However, the expression of *dicB* in the regulatory circuit of septation [25] was increased in cells expressing pCnuK9E, whereas expression of all other genes tested in the circuit, including *minC* and *minD,* did not show much change (Fig. 4A). These data suggest that the increased expression of the DicB protein caused by CnuK9E may lead to a negative effect on septation, which, in turn, induces filamentous growth.

The *dicB* gene is negatively regulated by the transcription factor DicA [26]. Thus, we investigated the expression of the *dicA* gene and of genes regulated by DicA using RT-PCR at 37°C [27]–[29]. The results showed that only in the CunK9E-expressing strain were the levels of *dicB*, *dicC, dicF,* and *ydfA* expression all elevated, whereas *dicA* expression was decreased (Fig. 4B). DicB is a cell division inhibition protein [26]. Furthermore, *dicF* RNA was reported to interfere with *ftsZ* mRNA translation [30]. These previous reports and our RT-PCR data (Fig. 4B) suggest that *dicA* downregulation leads to the upregulation of genes that negatively affect cell division. A mutant allele of *dicA*, *dicA1* is also known to cause filamentous growth in *E. coli* only at 37°C, and the DicB protein was overexpressed in this strain as well at 37°C [26]. Because *dicA* downregulation only occurred in the CnuK9E-producing strain at 37°C, we concluded that the transcriptional downregulation of *dicA* at 37°C by CnuK9E was the primary cause of the filamentous growth of *E. coli* cells at 37°C.

### A 10-fold downregulation of *dicA* causes a 3,700-fold increase in *dicB* transcripts at 37°C

Next, we analyzed the expression of the *dicA*, *dicB,* and *dicC* genes (Fig. 5A) in the presence of CnuK9E at 25°C and 37°C in MG1655 (WT) and MG1655*hns*. Total RNA was prepared from exponentially growing cells of MG1655 or MG1655*△hns* carrying either pHL355 (vector control) or pCnuK9E. Specific cDNA from each gene was generated by reverse transcription and quantified using real-time quantitative PCR (Table 1). The amount of transcript in cells harboring pHL355 at 25°C was used as a control and set to 1. Quantitation of the experimental samples was described as the fold-difference relative to the control (Table 1). Results from the experiments performed at 37°C showed that in WT cells expressing CnuK9E, transcripts of *dicA* decreased 10-fold, whereas those of *dicB* and *dicC* increased about 3,700- and 700-fold, respectively. This increase of *dicB* and *dicC* expression was considerately less in the absence of H-NS: in MG1655*△hns*/pCnuK9E, *dicB* and *dicC* expression was increased 78- and 24-fold, respectively, whereas that of *dicA* was decreased by half (0.55). Given that MG1655*hns* cells harboring pCnuK9E grew normally at 37°C, a 78-fold increase in *dicB* expression was not enough to elicit filamentous growth. Taken together, these results confirm previous results [16] and the results shown in Fig. 4B, that *dicA* works as a repressor of *dicC* and *dicB*. These data suggest that both CnuK9E and H-NS are required to efficiently downregulate *dicA* and, thus, to cause filamentous growth at 37°C.

At 25°C, a 40- to 50-fold increase in *dicB* and *dicC* expression and a 0.7- to 0.6- fold decrease in *dicA* expression was observed in CnuK9E producing cells. Interestingly, these changes in gene expression were observed regardless of the presence of H-NS (Table 1). These data suggest that CnuK9E alone could downregulate *dicA* gene without H-NS, and that, importantly, the negative effect of CnuK9E seems to be different at the two temperatures. Though *dicA* expression is downregulated in both MG1655/pCnuK9E and MG1655*△hns*/pCnuK9E cells at 37°C, by 0.1 and 0.55 fold, respectively, this causes large differences not only in cell physiology, filamentous vs. normal growth, but also in *dicB* expression, 3,700- vs. 78-fold increases, respectively. This suggests a complicated molecular mechanism in the regulation of *dicA* gene expression, perhaps quite unique.

### DicA binds to a putative operator sequence in the promoter region of *dicA*

In order to analyze the promoter of *dicA*, we needed to clone the promoter region. But the numbers of transformants after several cloning experiments (done in MG1655 strain) was unusual; in each time, we were getting none or a couple of transformants instead hundreds, suggesting that promoter DNA fragment of 122-bp cannot be cloned. Though, we tried the cloning in all available high-copy number plasmids in the laboratory and at different temperatures, we always got the same results of none but sometimes a few transformants. Thus, we sequenced the promoter region from some of the transformants, and found that each clone bears a single base change clustered at a putative operator region named Oc [28]. For example, an adenine to cytosine change at a specific residue occurred independently four times (Fig. 5B). We reasoned that this operator site is where DicA binds and the mutations prevented DicA from binding. DicA binding to the cloned promoter DNA in a high-copy plasmid might have caused a decrease in the effective DicA concentration, which, in turn, resulted in derepression of *dicB*. It is possible that over-expression of the DicB protein would have caused the same phenotype (lack of colony formation on agar plates) that CnuK9E did. Therefore, we cloned the promoter DNA fragment onto a single-copy pBAC-derived plasmid without mutation in the Oc site. Results using this plasmid suggested that DicA binds to Oc (Fig. 5B). The Oc sequence (20 nucleotides) is an inverted repeat that is typical of most operator sequences where homo-dimeric repressors bind.

An *in vivo* binding assay system was set up to assay DicA binding to Oc. The Oc sequence was cloned as an operator upstream of the *rpsL* gene under control of the *ant* promoter in the low-copy number plasmid used to measure Cnu-H-NS binding to the Ori-14 sequence, as shown in Fig. 1. This plasmid is referred to as pHL1105 (Fig. 1). If DicA or other proteins bound to Oc, the host strain HB101 would become resistant to streptomycin (Sm). We removed the *dicB* and *dicF* genes from HB101 to prevent cells from growing in filamentous form when the DicA protein concentration decreased due to binding to Oc. The resulting strain was named HL100. To assay the extent of Oc binding, we measured the growth of HL100 harboring pHL1105 with and without Sm in LB liquid medium. The ratio of the growth rates without/with Sm was calculated. This ratio, termed ‘GR’ and presented in Table 2, showed that the GR of HL100/pHL1105 at 37°C was 0.13. The GR of 0.13 indicated that, albeit very slowly, HL100/pHL1105 grew in the presence of Sm, suggesting transcription from the *ant* promoter was repressed by the binding of intracellular protein factors to the Oc operator. We anticipated that one of the factors was the DicA protein because HL100*△dicA*, which is identical to HL100 but without the *dicA* gene, did not grow in Sm medium if it harbored pHL1105 (Fig. 6B, Table 2). The data showing that DicA binds to Oc was strengthened by the following *in vivo* experiment. If the DicA protein was supplied from a high-copy number plasmid (pDicA, Fig. 1), the growth of HL100*dicA* in Sm medium became almost identical to that in Sm-minus medium, a GR of 0.97 (Fig. 6C, Table 2). Real-time quantitative PCR analysis showed that there was 65 times more *dicA-*specific mRNA in HL100*△dicA*/pDicA than in HL100/vector control (data not shown). Thus, it is likely that the higher levels of DicA protein from pDicA resulted in better Oc binding which caused a more efficient repression of the *ant* promoter, increasing the GR from 0.13 to 0.97.

We attempted an *in vitro* DicA-binding assay. The open reading frame of the *dicA* gene was cloned in a His-tag based plasmid (pHis-DicA, Fig. 1). The DicA protein was expressed only in the presence of IPTG (Fig. S2). However, most DicA protein was insoluble, leaving us an option to perform electrophoresis mobility shift assay (EMSA) using crude protein preparation (lane 5 Fig. S2). The EMSA was performed with a 90-bp DNA fragment containing the *dicA* promoter region (Fig. 5 B). Increasing amount crude protein in the assay yields a specific band of shifted mobility (arrow) only in the lanes where DicA protein is expressed (Fig. 7). These data indicated that DicA binds specifically to the dicA promoter region. However, the bands (star) that appear regardless the presence of dicA protein suggest that there is a protein (s) that binds to the *dicA* promoter region.

The following experiments demonstrated the sequence-specific binding of DicA to Oc. When the Oc of pHL1105 was replaced with Oc*, which contains a single base substitution (A to C with 4 independent incidents in Fig. 5B), HL100 harboring this plasmid (pHL1105*) did not grow in Sm medium (Fig. 6D, Table 2), suggesting that a single base change in Oc could abolish DicA binding. Note that the adenine to cytosine change in Oc* was observed as one of the mutations allowing the cloning of the *dicA* promoter region. There are two other operator sequences identified solely by nucleotide sequence, Om and Oa, in the *dicA* promoter region [16]. These two operator sequences were cloned into the *rpsL* plasmid and subjected to the *in vivo* DicA binding assay. Results indicated that DicA bound neither Om nor Oa (data not shown), indicating that DicA binds Oc in a sequence-specific manner.

### DicA binding to Oc is better at 25°C than at 37°C

The filamentous growth caused by *cnuK9E* (this study) or *dicA1* [26] depends on temperature; cells demonstrate normal growth at 25°C but filamentous growth at 37°C. Therefore, we tested the temperature-dependency of DicA binding to Oc. The GR of HL100/pHL1105 at 25°C was 0.67 (Fig. 6E, Table 2) and at 37°C it was 0.13 (Fig. 6A, Table 2). In addition, the mRNA concentration of *dicA* is 1.7 times higher at 37°C than at 25°C in WT cells (Table 1). Thus, it is likely that DicA binds to Oc better at 25°C than at 37°C. It should be emphasized that the lower binding activity of DicA to Oc at 37°C does not lead to filamentous growth in WT cells. However, the lower binding activity of DicA to Oc at 37°C might be the cause of the filamentous growth in CnuK9E-producing cells only at 37°C (see below).

### DicA bound to Oc enables *dicA* transcription but inhibits *dicC* transcription

The similarity of nucleotide sequence and putative gene structure of *dicA* and *dicC* to those of the immunity region of the lambdoid phages suggested that the promoter region that lies between the two genes (*PdicAC*) could function as a bipartite promoter that works like a genetic switch: either *dicA* or *dicC* could be turned on but not both at the same time [16]. The consensus promoter sequence for the sigma70-RNA polymerase of *dicC* was readily identified as shown in Fig. 5B. We identified the 5′-end of the *dicC* mRNA by 5′RACE assay (Fig. S3). The transcription initiation site is indicated as “+1 for *dicC*” in Fig. 5B. The invariant −11 adenine residue [31] in the −10 sequence of the *dicC* promoter exists exactly at the −11 position from the experimentally identified transcription initiation site of *dicC*, evidence that the *dicC* promoter works as depicted in Fig. 5B. The position of Oc that overlaps with the −10 sequence of the *dicC* promoter suggests that DicA binding to Oc could inhibit transcription from the *dicC* promoter. The repression of *dicC* by DicA has been demonstrated by others [16] and in Table 1 of this study. The expression of *dicC* increased 700-fold when *dicA* expression is 1/10th of normal.

Nucleotide sequence analysis of the *PdicAC* region did not reveal a consensus sequence for a promoter of *dicA*, suggesting that the *dicA* promoter is very weak and may need a transcriptional activator to recruit RNA polymerase to the promoter. Like the lambda repressor bound to the operator O_R_1 [32], we postulated that DicA bound to Oc is the transcriptional activator for *dicA*. This was tested as follows: a DNA fragment containing the *PdicAC* region (Fig 5B) was cloned in front of a promoterless kanamycin-resistant gene (*aph*) allowing the *dicA* promoter to drive the transcription of *aph*. This plasmid was named pHL1125 (Fig. 1). The half-maximal inhibitory concentration for kanamycin (IC_50_kan) of the different host cells harboring pHL1125 was measured as an assay for transcription from the *dicA* promoter (See materials and methods). The results are summarized in Table 3. HL100 cells harboring pHL1125 showed an IC_50_kan of 73 µg ml^−1^ at 37°C. The IC_50_kan of the negative controls for *dicA* transcription, HL100/pHL1124 (no *PdicA*) and HL100*△dicA*/pHL1125 was about 4 µg ml^−1^ at 37°C. Therefore, the results for HL100/pHL1125 suggested that considerable transcription is initiated from the *dicA* promoter (Table 3). The lack of transcription from HL100*△dicA*/pHL1125 is an interesting result, because it suggests that without DicA protein available, there is no transcription from the *dicA* promoter. Thus, DicA appears to be a transcriptional factor required for transcription initiation. When DicA protein was supplied from a plasmid (pDicA), HL100*△dicA*/pHL1125 exhibited an IC_50_kan of 36 µg ml^−1^ at 37°C (Table 3). It is not clear why the IC_50_kan decreased to 36 (from 73) when DicA protein was supplied from a plasmid. We assumed that higher concentration of DicA might have caused more insoluble DicA (as shown in Fig. S3), and that would have decreased the effective concentration of DicA. Nevertheless these data suggest that DicA functions as a transcriptional activator for *dicA* and a transcriptional repressor for *dicC*.

We also measured the effect of temperature on *dicA* transcription *in vivo*. IC_50_kan of HL100/pHL1125 was measured independently three times at the two temperatures, 25°C and 37°C. These measurements revealed that HL100/pHL1125 grown at 25°C always showed a higher IC_50_kan than at 37°C (Table 3). These data suggest more transcription of *dicA* at 25°C than at 37°C, though there is 70% less *dicA* transcript at 25°C (Table 1),consistent with our data showing better binding of DicA to Oc at 25°C than at 37°C.

### CnuK9E antagonizes DicA binding to Oc

Our data indicate that transcriptional downregulation of *dicA* by CnuK9E likely causes filamentous growth and that DicA binding to Oc activates *dicA* transcription. We reasoned that CnuK9E exerts a negative effect on DicA binding to Oc and downregulates *dicA*, which, in turn, causes filamentous growth. To test this, DicA binding to Oc was assayed in the presence of varying amounts of CnuK9E. The GR of HL100*△dicA*/pHL1105 harboring pDicA was shown to be 0.97 and that of HL100/pHL1105 was 0.13 (Table 2), suggesting that the higher concentration of DicA due to the pDicA plasmid resulted in more DicA binding to Oc, which resulted in nearly 100% repression of the *ant* promoter. If CnuK9E antagonizes DicA binding to Oc, the GR of HL100*△dicA*/pHL1105/pDicA harboring pCnuK9E should become less than 0.97 as the IPTG concentration of the growth medium increases. To perform this experiment, we inserted the *dicA* gene in pHL1105, creating pHL1191 (Fig. 1). The GR of HL100/pHL1191 was the same as that of HL100*△dicA*/pHL1105/pDicA. Thus, we used HL100/pHL1191 to measure the antagonistic effect of CnuK9E on DicA binding.

The growth of HL100/pHL1191/pCnuK9E was measured using different concentrations of IPTG at the two different temperatures in LB medium containing Sm; the results are shown in Fig. 8. We presented growth instead of GR in Fig. 8 because the growth rate changed with time at the effective IPTG concentrations; thus, we could not measure GR in these cases. However, by comparing growth rates with controls (no IPTG or no Sm), we were able to measure the antagonizing effect of CnuK9E on DicA binding activity. As IPTG concentration increased, growth of HL100/pHL1191/pCnuK9E decreased at both temperatures (Fig. 8 A and B). These data demonstrated that CnuK9E antagonizes DicA binding to Oc. The concentration of IPTG that resulted in less growth than the control (no IPTG or no Sm) was 60 µM at 25°C and 20 µM at 37°C, suggesting that the antagonizing effect of CnuK9E on DicA binding was more efficient at 37°C. This is presumably because DicA binds to Oc better at 25°C than at 37°C (Fig. 6E), or that CnuK9E and/or temperature cause structural changes in DNA.

Because CnuK9E downregulated *dicA* expression better in the presence of H-NS (Table 1) and CnuK9E can form a protein complex as efficiently as its wild-type Cnu with H-NS (Fig. S1), it may be the CnuK9E-H-NS complex that antagonizes DicA binding to Oc. We tested this possibility by repeating the growth measurement at 37°C in HL100*hns*, where H-NS is not present (Fig. 8C). In this case, the effective concentration of IPTG that allowed less growth than the control was 40 µM (Fig 8C), higher than the 20 µM observed when H-NS was present in HL100/pHL1191/pCnuK9E (Fig. 8B). These results demonstrate that more CnuK9E is required to antagonize DicA binding in the absence of H-NS and imply that the CnuK9E-H-NS complex antagonizes DicA binding to Oc more effectively.

### DicA is homologous to RovA/SlyA in structure and function

A homology search of the protein data base and amino acid sequence comparisons using Protein Blast [33] indicated that the N-terminal 70 amino acid residues (1–70), of DicA have significant matches with the N-terminus of the C2 protein (1–68) of the *Salmonella* phage P22 (Fig. 9). This has also been demonstrated previously [16]. The homology study also revealed that the C-terminal portion of DicA (80–135) shares a number of matches with the N-terminal portion (25–81) of RovA (*Yersinia*) and SlyA (*Salmonella*), as presented in Fig. 9. Amino acid alignment indicated that the N-terminal portion of DicA and C2 share 59% identical (37/63) and 78% related (49/63) amino acids (Fig. 9). The C-terminus of DicA shares 29% identical (16/55) and 44% related (24/55) amino acids with the N-terminal region of RovA and SlyA (Fig. 9). RovA and SlyA both have a winged helix domain at the N-terminus that binds DNA [34], [35]; the SlyA dimer makes contact with a specific DNA sequence [36]. As was noted by Bejar et al (1986), the nucleotide sequences of *dicC*, *PdicAC*, and the 5′-half of *dicA* show substantial similarity to the *immC* region of P22 phage. The C-terminus of DicA has extensive homology with RovA and SlyA and not with the C-terminus of the C2 protein, indicating that DicA acquired the function of the C-terminus after the integration event of the Kim/Qin phage into the *E*. *coli* chromosome. Why the *dicA* gene evolved to acquire this functional domain is not completely understood.

The functional similarity between DicA and RovA/SlyA is notable; RovA and SlyA are transcription activators for a group of pathogenic genes [35], [37]. The DNA-binding activity of RovA depends on temperature and is better at 25°C than at 37°C [38]. RovA makes contacts with the RNA polymerase of *E. coli* to enhance transcription [35], thus is a direct transcription activator, but at some promoters RovA and SlyA enhance transcription by reversing the silencing imposed by H-NS [39], [40], thus activity is indirect. The antagonizing effect of the CnuK9E-HNS complex on DicA binding suggests complicated binding dynamics between DicA and the Cnu-HNS complex. Our data (Table 1) showed that transcription from *dicA* increases when H-NS is absent. We are currently investigating the role of H-NS and Cnu in the regulation of *dic* genes.

## Supporting Information

Figure S1 **Elution profile of His-Cnu and His-CnuK9E from a Nickel-affinity column.**
(TIF)

Figure S2 **Overproduction of DicA protein.**
(TIF)

Figure S3 **Identification of 5′-end of the** ***dicC*** **transcript.**
(TIF)

Table S1 **Primers used in this study.**
(DOCX)
