# Supplementary figures and images for: The CnuK9E H-NS Complex Antagonizes DNA Binding of DicA and Leads to Temperature-Dependent Filamentous Growth in E. coli
Source: PLoS One. 2012 Sep 13;7(9):e45236. doi: 10.1371/journal.pone.0045236 (PMC3441716; doi:10.1371/journal.pone.0045236)

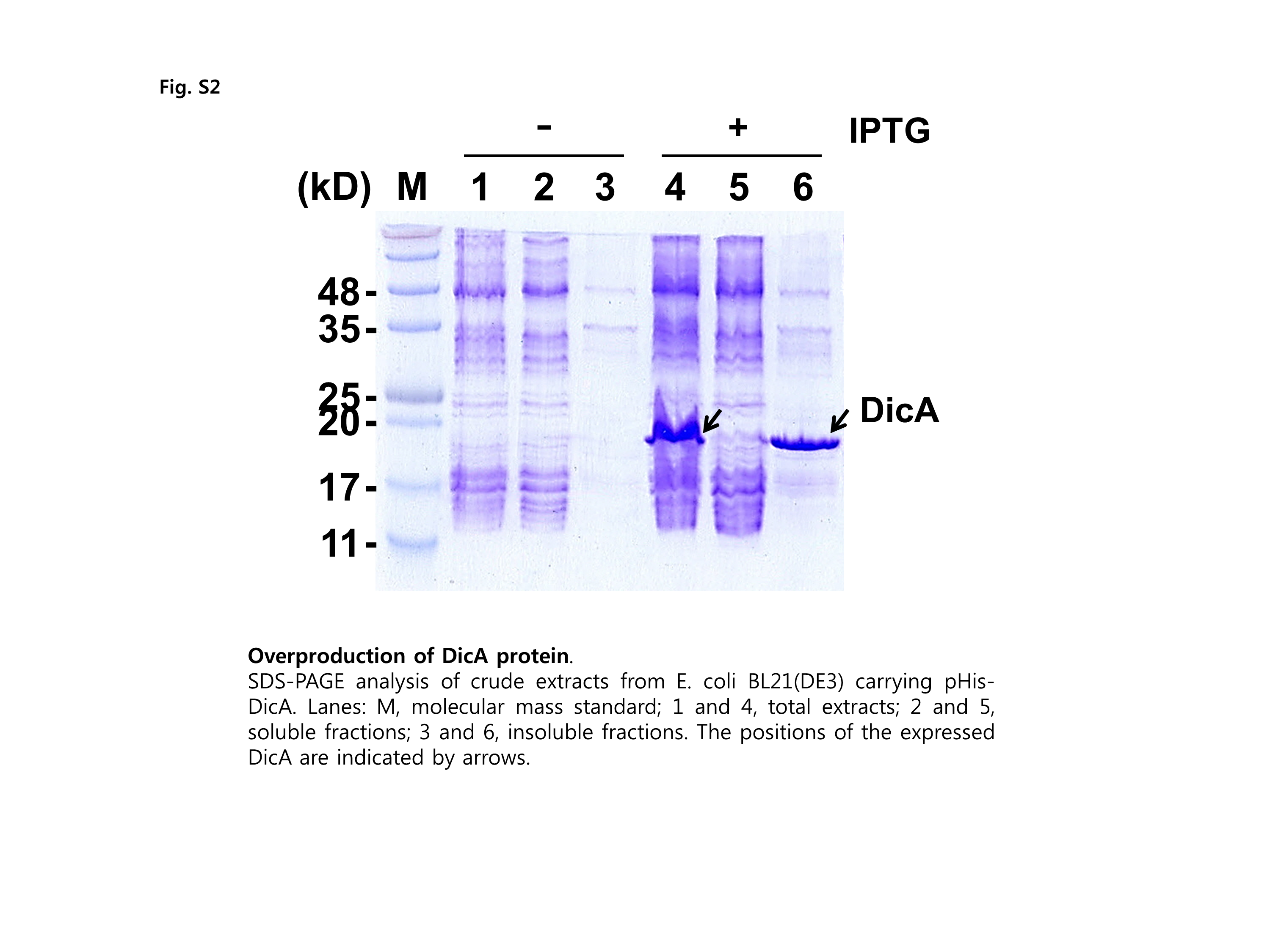

Supplement: Figure S2 — Overproduction of DicA protein. (TIF) [file pone.0045236.s002.tif]
