# Supplementary material for: The CnuK9E H-NS Complex Antagonizes DNA Binding of DicA and Leads to Temperature-Dependent Filamentous Growth in E. coli
Source: PLoS One. 2012 Sep 13;7(9):e45236. doi: 10.1371/journal.pone.0045236 (PMC3441716; doi:10.1371/journal.pone.0045236)
Supplement: Table S1 — Primers used in this study. (DOCX) [file pone.0045236.s004.docx]

**Table S1. Primers used in this study**

| **Name** | **Sequence (5’-3’)** | **used** |
| --- | --- | --- |
| Ori14-up | ATGATCGGTGATCCTG | Operator hybrid |
| Ori14-down | CAGGATCACCGATCAT |  |
| Oc-up | TTGTTAGTCATAACTAACAA |  |
| Oc-down | TTGTTAGTTATGACTAACAA |  |
| Oc*-up | TTGTTAGTCATAACTCACAA |  |
| Oc*-down | TTGTGAGTTATGACTAACAA |  |
| pHL355-RM-F | TTCACACAGGAAACAGAATTC | Cnu mutagenesis |
| pHL355-RM-B | CATCCGCCAAAACAGAAGCTT |  |
| ftsZ-RT-F | TGGAACTTACCAATGACGC | RT-PCR |
| ftsZ-RT-B | CCAACCGCTGTTTTACG |  |
| minE-RT-F | ATGGCATTACTCGATTTCTTTC |  |
| minE-RT-B | GAATATCTTTACGCAACTGCG |  |
| minD-RT-F | GCATTATTGTTGTTACTTCGG |  |
| minD-RT-B | TAATCAGGTCGAGATTACGC |  |
| minC-RT-F | CAAACACGCCAATCGAG |  |
| minC-RT-B | ACTGACGTTGAGTACAACGG |  |
| sfiA-RT-F | GCGTCATCATGGGCTG |  |
| sfiA-RT-B | ATGTACACTTCAGGCTATGCAC |  |
| dicB-RT-F | ATGAAAACGTTATTACCAAACG | RT-PCR and Real-Time-qPCR |
| dicB-RT-B | GTAAACGAGCCAGCATTG |  |
| dicA-RT-F | GGAAACAAAAAATTTAACTATCG |  |
| dicA-RT-B | TCACTATCACCCCGTTCC |  |
| dicC-RT-F | CTTAAAACTGACGCTCTTTTGT |  |
| dicC-RT-B | GATGCCTCCTGTAGACGC |  |
